# Engineering of the Melanoma Inhibitor of Apoptosis (ML‐IAP) Anticancer Peptide Through Comprehensive In Silico Approaches

**DOI:** 10.1155/humu/9356098

**Published:** 2026-04-10

**Authors:** Haitham Ahmed Al-Madhagi, Muhammad shahab, Zheng Guojun, Gamil Al-Madhagy, Mohammed Bourhia, Gamal A. Shazly, Musaab Dauelbait, Zunnan Huang

**Affiliations:** ^1^ Biochemical Technology Program, Thamar University, Dhamar, Yemen, thuniv.net; ^2^ Dongguan Key Laboratory of Computer-Aided Drug Design, Guangdong Provincial Key Laboratory for Research and Development of Natural Drugs, School of Pharmacy, Guangdong Medical University, Dongguan, Guangdong, China, gdmu.edu.cn; ^3^ State Key Laboratories of Chemical Resources Engineering, Beijing University of Chemical Technology, Beijing, China, buct.edu.cn; ^4^ Department of Oral and Maxillofacial Surgery, University of Damascus, Damascus, Syria, damascusuniversity.edu.sy; ^5^ Laboratory of Biology, Polydisciplinary Faculty of Safi–Cadi Ayyad University, Safi, Morocco, uiz.ac.ma; ^6^ Department of Pharmaceutics, King Saud University, Riyadh, Saudi Arabia, ksu.edu.sa; ^7^ Department of Scientific Translation, University of Bahri, Khartoum, Sudan, bahri.edu.sd

**Keywords:** docking, MD simulation, melanoma, PCA, PPI, saturation mutagenesis

## Abstract

**Background and Aims:**

Melanoma represents the most malignant type of skin cancer. It is estimated that approximately 100,000 new cases of melanoma were diagnosed in 2022, resulting in over 7600 deaths in the United States alone. Recently, anticancer peptides (ACPs) have emerged as novel therapeutic agents for cancer, offering higher potency, biocompatibility, and fewer adverse reactions in host cells. One of the druggable targets of melanoma is the melanoma inhibitor of apoptosis (ML‐IAP), conventionally inhibited by the nonapeptide AVPIAQKSE. The current study is aimed at enhancing both the binding affinity and safety profile of this peptide through in silico peptide engineering.

**Methods:**

Initially, the 3D structure of the protein was downloaded from the Protein Data Bank (PDB) (ID: 1OXQ) and prepared. The hotspot residues at the interface were detected using Discovery Studio Client 2021. Afterwards, saturation mutagenesis was conducted to discover the best potential amino acid substitutions with a positive impact on the binding affinity. The lead candidates were docked to the receptor via HPEPDOCK 2. Additionally, the safety profile was assessed using the ToxIBTL and AllerCatPro 2 servers. Finally, molecular dynamics simulations and principal component analysis were performed to check the stability of the best complexes.

**Results:**

HVPIAQKSE, WVPWAQKSE, and HVPWAQKSE were the best mutants that could be superior to the original peptide in terms of binding affinity as well as safety profile. MD results confirmed the stability, flexibility, reduced local motions, conformational changes, and more compact structure upon binding the receptor for 200 ns, which deserve in vitro validation as a better melanoma ACP therapeutic option.

**Conclusion:**

These variants displayed increased flexibility, reduced conformational alterations and local motions, and a more compact configuration, suggesting greater stability compared with the reference peptide.

## 1. Introduction

It has been estimated that around 100,000 new melanoma cases were diagnosed in 2022, resulting in > 7600 deaths in the United States alone. The spread of melanoma is recorded to be the fastest among all types of cancers. Invasive melanoma is the least prevalent form of skin cancer, accounting for about 1% of all skin cancer cases, but it is the fifth deadliest type of cancer affecting both genders [1, 2]. On a global scale, melanoma rates were 3.4 new cases and 0.55 deaths per 100,000 people in 2020. Australia and New Zealand exhibited the highest rates, attributed in part to factors such as smoking, drinking, poor diet, obesity, and metabolic diseases. In Australia, it reaches a rate of 60 new cases per 100,000 [1]. Incidence of melanoma increased from 30.3 to 72.1 per 100,000 person‐year between 2000 and 2022 [2].

Although more countries in Europe observed an increase in cancer cases, overall, cancer‐related deaths were declining. A significant rise in new cases was noted among individuals over 50, irrespective of gender [3]. The positive development lies in the declining incidence of melanoma, credited to the effectiveness of immunotherapeutic and targeted therapy. Unlike radiation and chemotherapy, advanced therapeutics have the capability to activate and redirect the dormant immune system, enabling it to combat cancerous cells in a more targeted manner, thus reducing adverse effects on the host. These treatment options indeed contributed to an approximate 4% decrease in the incidence of melanoma [4]. The most prevalent phenotypic risk factor for melanoma is having skin that is prone to sunburn. This is true as some skins with low melanin have higher penetration of sun irradiation that would result in skin damage and transformation into cancer. The most substantial genotypic risk factors are inherited variants of the melanocortin‐1 receptor (MC1R). Individuals with a high number of common nevi, large congenital nevi, multiple nevi, or atypical nevi (dysplastic nevi) are at increased risk [5]. The most critical risk factor of invasive melanoma involves UV irradiation, with intermittent, high exposure to the sun playing a particularly significant role [6–9]. One of the primary suppressors preventing melanoma cells from initiating apoptosis is the melanoma inhibitor of apoptosis (ML‐IAP). ML‐IAP belongs to the IAP protein family and is known to be upregulated in various types of tumors [10]. IAPs constitute a conserved protein class characterized by the incorporation of one to three baculoviral inhibitor of apoptosis repeat (BIR) domains, pivotal motifs facilitating protein–protein interactions. These BIR domains feature zinc‐coordinated architectures reinforced by conserved cysteine and histidine residues, serving as essential structural elements enabling IAPs to effectively interact with a diverse spectrum of proteins, particularly caspases [11]. Eventually, ML‐IAP prevents the suicide of melanoma cells and, thus, is a leading factor of melanoma survival, progression and resistance to radiation. Hence, it was a hotspot for targeted chemotherapy and immunotherapy in the recent decade [12]. Currently, numerous new chemical entities have demonstrated their potency against melanoma, which include imidazole derivatives, benzimidazole derivatives, imidazothiazole derivatives, quinoline derivatives, pyrazole pyrimidines derivatives, indole derivatives, and heterotricyclic derivatives [13]. Nonetheless, the serious side effects of most of the classical antitumor chemical compounds represent an obstacle for their successful translation into a clinical setting. In recent years, anticancer peptides (ACPs) are emerging as more powerful weapons against wide types of cancers with safer toxicity profiles. By looking at the function and mechanism of action of these peptides, it is important to optimize new peptide inhibitor. ACPs operate by creating pores in cell membranes, a process that can initiate necrosis or apoptosis in abnormal cells [14–16]. Despite its demonstrated potency as an apoptosis‐inhibition blocker, the original ACP AVPIAQKSE exhibits certain limitations, including moderate binding affinity toward ML‐IAP and concerns regarding its potential toxicity and stability in physiological environments [17]. These challenges may restrict its therapeutic potential and impede further clinical development as an anticancer agent. To address these limitations, the present study was designed with the goal of enhancing both the binding affinity and safety profile of AVPIAQKSE. We hypothesized that rational, in silico engineering of AVPIAQKSE could produce peptide variants exhibiting improved ML‐IAP interaction and reduced predicted toxicity. Specifically, this work focuses on evaluating whether structure‐based computational design strategies can generate AVPIAQKSE derivatives with enhanced binding affinity to ML‐IAP, while also assessing if bioinformatics screening can identify peptide variants with reduced predicted toxicity and improved stability. To achieve these aims, we employed a comprehensive suite of bioinformatics and molecular modeling approaches to systematically optimize AVPIAQKSE as a potential antimelanoma therapeutic.

## 2. Methods

### 2.1. Design of Methodology

**Figure 1:**
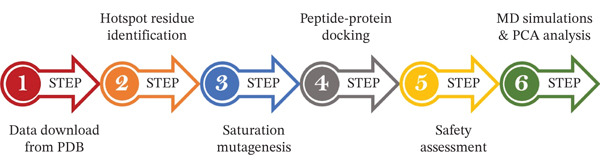
The strategy summary deployed in the present study to achieve the mentioned goals.

The strategy deployed to accomplish the goals of our study is shown in Figure 1.

### 2.2. Retrieval of Target Protein Structure

The three‐dimensional structure of the ML‐IAP–peptide complex was retrieved from the Protein Data Bank (PDB) (ID: 1OXQ) [17]. This structure, solved by x‐ray crystallography at a resolution of 2.30 Å with an R‐free of 0.218, was expressed in *Escherichia coli* BL21 (DE3). The PDB file comprises five identical chains; only Chain E, representing ML‐IAP, was utilized for further computational analysis. Prior to docking, polar hydrogens and atomic charges were assigned using UCSF Chimera with the AMBER ff19SB force field. Chain F, corresponding to the inhibitory peptide, lacked five C‐terminal residues in the deposited structure. These residues were reconstructed using Modeller‐based loop modeling integrated within Chimera, and the complete peptide structure was subjected to energy minimization to relieve steric clashes and optimize geometry. The resulting model was energy‐minimized to ensure proper geometry and to resolve any steric clashes. The modeled peptide–protein complex was visually inspected for structural integrity, with particular attention paid to the orientation and interactions of the rebuilt segment. It is noted that, based on the crystal structure, the primary interaction interface is mediated by the first four N‐terminal residues of the peptide. The prepared, energy‐minimized complex was then used as the input for all subsequent hotspot identification, mutagenesis, and docking protocols.

### 2.3. Peptide Hotspot Residues Identification and Mutagenesis

The processed PDB file was submitted to the mCSM‐PPI2 online platform (https://biosig.lab.uq.edu.au/mcsm_ppi2/) [18] using default parameters. Interface hotspot residues between the protein and the inhibitory peptide were identified based on predicted changes in binding free energy (*Δ* *Δ*G) upon alanine scanning, as calculated by the platform. Residues with predicted destabilizing effects greater than −1.0 kcal/mol upon alanine substitution were considered significant hotspots. Subsequently, each identified hotspot residue was subjected to computational saturation mutagenesis using the same server, generating all possible single‐point amino acid variants at these positions. All mutations were evaluated by mCSM‐PPI2 for their predicted effect on binding affinity (*Δ* *Δ*G), and only those substitutions predicted to improve or maintain binding were shortlisted for further study. Predicted effects of mutations were cross‐validated, where possible, with previously reported mutational data from the literature. This two‐step procedure ensured that only those peptide variants with the most favorable predicted binding profiles were selected for subsequent protein–peptide docking experiments.

### 2.4. Protein–Peptide Docking

The top‐ranked peptide candidates were then assessed for binding potential using the HPEPDOCK 2 online server (http://huanglab.phys.hust.edu.cn/hpepdock/) [19]. For each docking, the receptor structure was uploaded, and peptide candidates were submitted as amino acid sequences in single‐letter code. HPEPDOCK 2′s built‐in modeling algorithm was used to generate peptide structures de novo from the provided sequences. All other docking parameters were maintained at their default settings as specified by the server documentation, with no manual adjustments. To validate the docking protocol, the original wild‐type (WT) peptide was redocked using the same procedure, and the resulting complex was compared with the experimental structure by calculating the root‐mean‐square deviation (RMSD). Docking results for all candidates were ranked according to HPEPDOCK 2 global docking score. The top‐ranked poses for each peptide were visually inspected to confirm plausible binding modes and consistency with known interaction sites. Negative controls or decoy peptides, consisting of randomized sequences, were also docked to evaluate the specificity of the protocol; these produced consistently lower docking scores than the native and designed peptides.

### 2.5. Redocking

The original peptide (WT) was subjected to redocking process to ensure the validation and accuracy of the molecular docking output of the HPEPDOCK 2 server (details are provided above). Afterwards, the two generated models, that is, the original as well as the redocked models were superimposed via Pairwise Structure Alignment online tool and choosing the alignment method as flexible. The RMSD is an indicative of the accuracy of resemblance of the outputted models (the lower it is, the more accurate the docking is).

### 2.6. Molecular Dynamics (MD) Simulations

To explore the dynamic behavior of both the WT complex and the designed peptide, we utilized MD simulations. We initiated our simulations by setting up the system topology and coordinates using the tLEAP module of Amber v.22 [20–22]. The ff19SB force field was applied for protein and peptide parameterization, and systems were solvated in a rectangular box of explicit TIP3P water molecules, extending 14.0 Å beyond the solute in all directions. System neutrality was achieved by adding sodium or chloride counter‐ions as needed [23–25]. Initial energy minimization was conducted in two stages: 4000 steps of steepest descent followed by 2000 steps of conjugate gradient minimization, ensuring relaxation of the solvent and removal of steric clashes. The system was then gradually heated from 0 to 298 K over 50 ps under constant volume conditions, employing harmonic restraints on heavy atoms. Equilibration was performed for an additional 100 ps at 298 K and 1 atm pressure under the NPT ensemble, with restraints gradually released. For each system, three independent production runs of 200 ns each were performed, with different initial random velocity seeds to ensure statistical reproducibility. All production simulations were carried out under periodic boundary conditions using the particle mesh Ewald method for long‐range electrostatics and a 2‐fs integration timestep. Covalent bonds involving hydrogen atoms were constrained using the SHAKE algorithm [26]. Trajectory analyses were conducted using CPPTRAJ and PTRAJ modules [27–29]. Backbone RMSD, radius of gyration (Rg), and C*α* atomic fluctuations (root‐mean‐square fluctuation [RMSF]) were calculated to assess structural stability and flexibility. Reported values represent averages over the three independent replicates, with standard deviations provided as measures of statistical uncertainty.

### 2.7. Safety Profile of the Generated Peptides

After demonstrating the potential enhancement of the engineered peptides, their possible safety profile was assessed. Toxicity using ToxIBTL tool [30] and allergenicity through AllerCatPro 2.0 webserver (https://allercatpro.bii.a-star.edu.sg/) [31] were deployed by inputting the peptide sequences setting while keeping other parameters as default.

## 3. Results and Discussion

### 3.1. PPI of the Original Peptide

**Figure 3:**
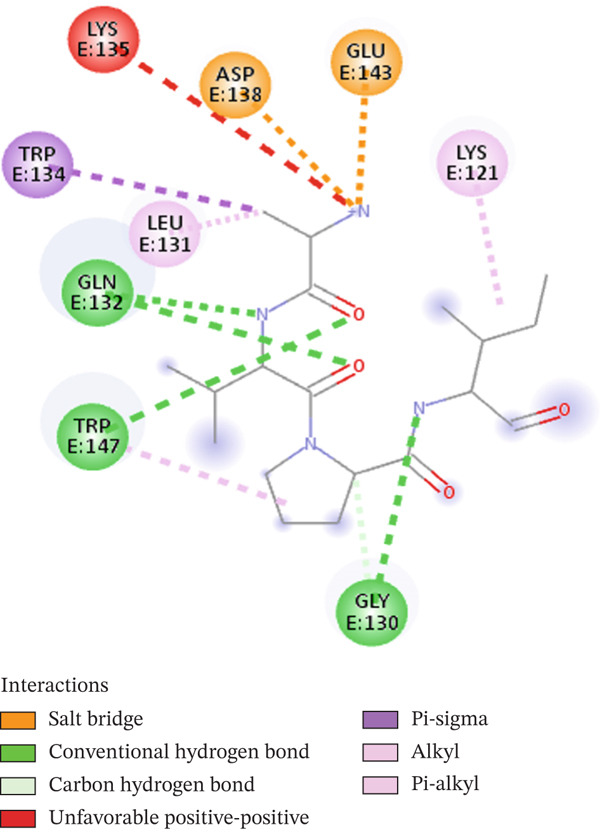
A 2D diagram ecludiating the differet types of the original peptide–receptor interactions.

Firstly, we display the whole complex to provide an bird′s‐eye view and then glimpse into the bonding pattern. The representation in Figure 2 shows that the WT peptide falls well within the active pocket of the receptor and was subjected to rigorous in silico mutagenesis. The type of interactions between native inhibitory peptide with the target protein was elucidated through a 2D diagram of the Discovery Studio Client 2021 software, as shown in Figure 3. Indeed, it turned out that almost all types of interactions and bonds were found, that is, H‐bonds with Gly 130, Gln 132, and Trp 147, salt bridges with Asp 138 and Glu 143, a Pi‐sigma bond with Trp 134, and one alkyl and pi‐alkyl with Lys 121 and Leu 131. Additionally, unfavorable positive–positive distraction was formed with Lys 135 and peptide N of Ala. This broad spectrum of bonds and interactions interprets the powerful inhibitory nature of the peptide to the target protein.

Figure 2Receptor–peptide complex visualized in (a) surface and space‐filling mode and in (b) cartoon and sticks configuration.

**Figure:**
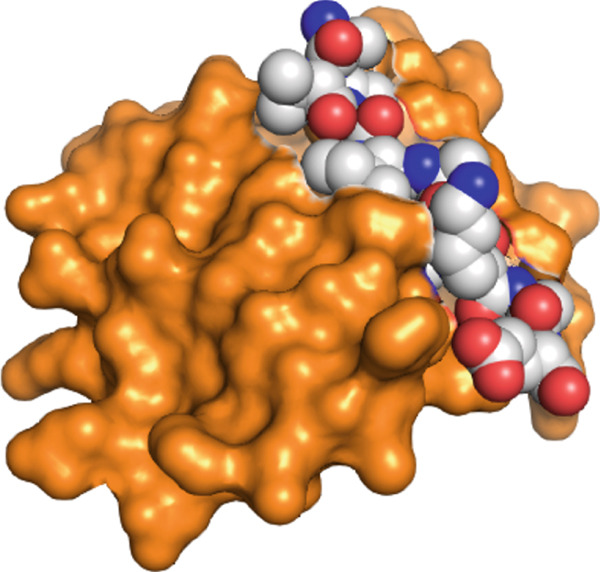
(a)

**Figure:**
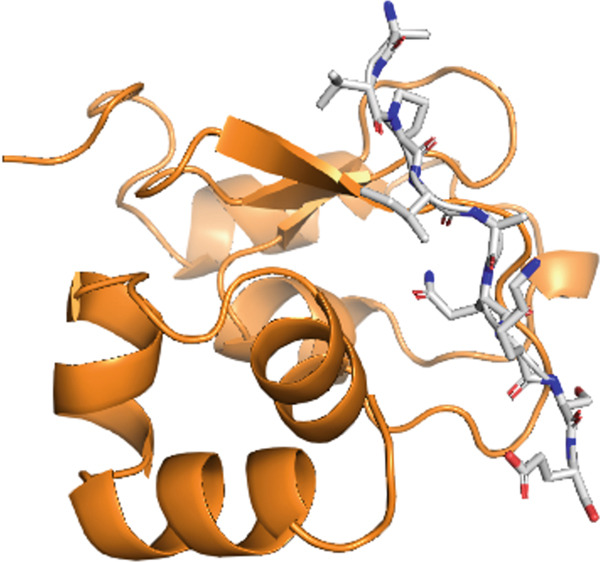
(b)

### 3.2. Redocking

**Figure 4:**
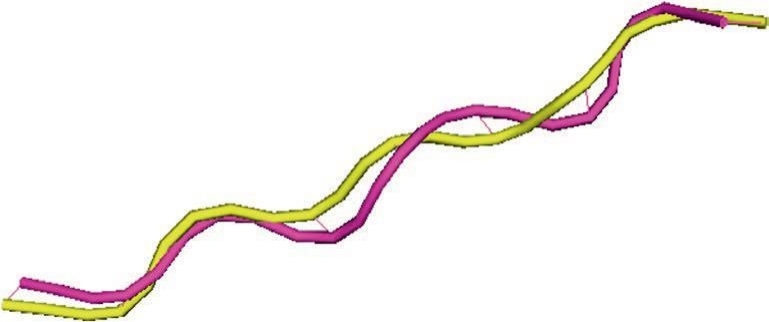
Strucutural alignment (superimposition) of the original (shown in magenta) and redocked (shown in yellow) WT peptide using flexible pairwise alignement.

To ensure the accuracy of the docking results, we performed redocking process of the WT peptide using the same server (HPEPDOCK 2) and compared them with the original version via structural superimposition. As shown in Figure 4, the two models are superimposed very well to each other, indicating the high accuracy and precision of the HPEPDOCK 2 server. Numerically, the RMSD of the superimposition was 1.20 Å, confirming the accuracy of the docking process.

### 3.3. Mutagenesis

**Figure 5:**
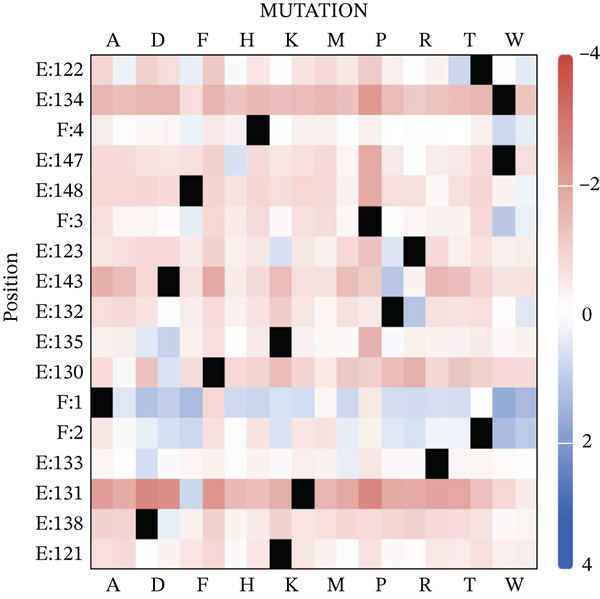
Saturation mutagenesis of the receptor–peptide complexes showing the most hotspot residues in the receptor as well as the peptide inhibitor. Chain E for receptor, whereas Chain F for peptide. The more negative (blue color) the replaced residue is, the better the enhanced binding is and vice versa regarding the red color. Black blocks indicate the same residue as the original one (mCSM‐PPI 2 server, https://biosig.lab.uq.edu.au/mcsm_ppi2/).

The mCSM‐PPI 2 server (https://biosig.lab.uq.edu.au/mcsm_ppi2/) is powerful in that it is capable of conducting saturation mutagenesis of the two interacting protein chains and their consequence on the overall affinity. In the present study, the server generated a heat map summarizing the most hotspot residues of all chains that have the most significant impact on the PPI as depicted in Figure 5.

Our results showed that Residue 1 was the most significant hotspot for mutagenesis. This is due to the fact that this residue is the nearest to the active center of the target protein. The mCSM‐PPI 2 algorithm ranked the bulky hydrophobic amino acids (Trp and Tyr) in place of the small native one, Ala. However, negatively charged residues such as Glu, once replaced A1, had a negative impact on the binding affinity of the peptide to the target protein (−117 kcal/mol), contrary to the mCSM‐PPI 2 prediction. In contrast, His, a positively charged amino acid, was the best in variant leading to enhanced binding affinity as confirmed by HPEPDOCK 2. The top ranked variants of the inhibitory peptide are listed in Table 1.

**Table 1:** Protein–peptide docking of the generated mutants along with the effect on binding affinity.

Wild‐type	Residue number	Mutant	Distance‐to‐interface (Å)	mCSM‐PPI 2 prediction	Affinity	HPEPDOCK 2 (kcal/mol)
						‐125
Ala	1	Trp	2.681	1.7	Increasing	−151
Ala	1	Tyr	2.681	1.398	Increasing	−147
Val	2	Trp	2.961	1.355	Increasing	−149
Pro	3	Trp	3.321	1.063	Increasing	−153
Ala	1	Glu	2.681	0.975	Increasing	−117
Ile	4	Trp	3.159	0.783	Increasing	−159
Ala	1	His	2.681	0.752	Increasing	−161

### 3.4. PPI of Mutants (MTs)

#### 3.4.1. Single MTs

**Figure 6:**
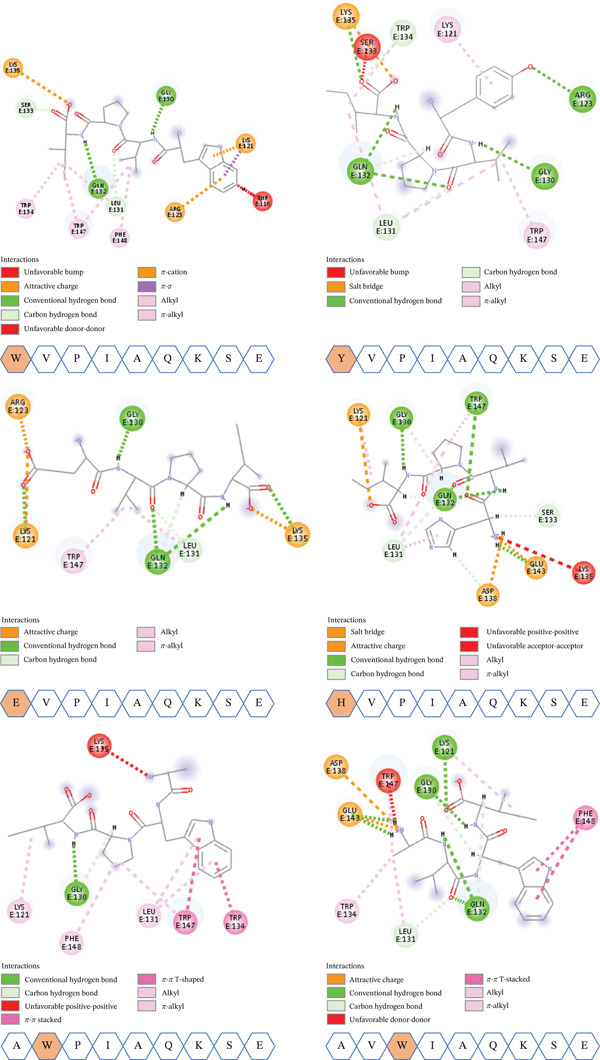
A 2D diagram elucidating the types of interactions of the generated single mutants with the receptor along with the substituted amino acids.

The protein–peptide interactions in 2D are shown in Figure 6. In a similar fashion to the native peptide, the MT also formed different types of interactions and bonds including H‐bonds, different VdW interactions, hydrophobic, unfavorable interactions (unfavorable bump and unfavorable donor–donor, unfavorable acceptor–acceptor, and unfavorable positive‐positive), and salt bridges. These account for the stronger binding energy between the mutated peptides and the target protein.

#### 3.4.2. Double MTs

**Figure 7:**
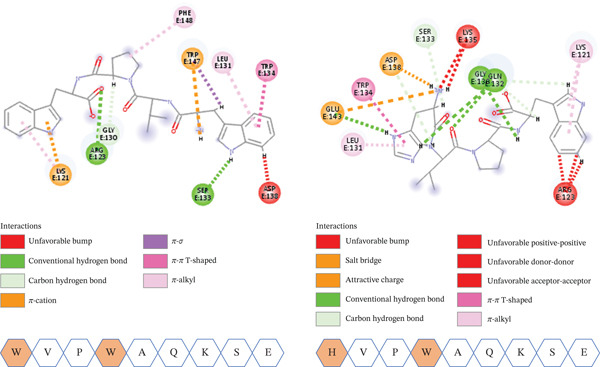
A 2D visualization elucidating the types of interactions of the generated double mutants with the receptor along with the substituted amino acids.

In addition to the singly mutated peptides, the best two variants that showed the highest binding affinity were combined to produce the most potentially potent peptide. Both HVPWAQKSE and WVPWAQKSE displayed stronger binding affinity (−167 vs. −173 kcal/mol) in comparison with the singly mutated and the original peptide versions. This was accomplished via a wide array of interactions involving H‐bonds, unfavorable bonds, salt bridges, VdW, and hydrophobic as illustrated in Figure 7.

#### 3.4.3. Safety Profile

After confirming the higher binding affinity of the MTs, the safety profile was also examined. It turned out that all of the tested variants were predicted to be safe in terms of immunogenicity, that is, they are nonallergen. On the other hand, three of the variants (MTs 2, 4, and 5) exhibited toxicity toward host cells as summarized in Table 2. Nevertheless, these results are predictions by bioinformatics that need further wet experiments validation.

**Table 2:** Allerginicity and the toxicity of the examined peptide mutants.

ACP	Allergenicity	Toxicity
Native peptide	Nonallergen	Nontoxic
WVPIAQKSE	Nonallergen	Toxic
YVPIAQKSE	Nonallergen	Nontoxic
AWPIAQKSE	Nonallergen	Toxic
AVWIAQKSE	Nonallergen	Toxic
EVPIAQKSE	Nonallergen	Nontoxic
AVPWAQKSE	Nonallergen	Nontoxic
HVPIAQKSE	Nonallergen	Nontoxic
WVPWAQKSE	Nonallergen	Toxic
HVPWAQKSE	Nonallergen	Nontoxic

#### 3.4.4. MD Simulations

##### 3.4.4.1. Protein Structural Stability Analysis.

To evaluate the structural stability of the ML‐IAP complexes with WT and engineered peptides, backbone RMSD was calculated over 200 ns of MD simulation for each system (Figure 8). In every comparison, the WT peptide complex (AVPIAQKSE) exhibited a gradual and continuous rise in RMSD, reaching values of approximately 4.5–5.0 Å by the end of the simulation. This pattern indicates the notable conformational drift, increased flexibility, and partial loss of binding stability for the WT complex, particularly in the later stages of the trajectory. In contrast, the MT peptide complexes demonstrated significantly enhanced stability. In Figure 8a, the HVPIAQKSE complex (red line) maintained RMSD values between 1.0 and 2.0 Å for the entire 200 ns, with minimal fluctuations after the initial equilibration phase (0–20 ns). Similarly, the HVPWAQKSE MT (Figure 8b) showed an RMSD plateau around 1.5 Å, with no substantial upward trend throughout the simulation. The WVPWAQKSE MT (Figure 8c) exhibited RMSD values stably between 1.5 and 2.0 Å, whereas the WVPWAQKSE double MT (Figure 8d) remained below 2.0 Å, confirming robust complex integrity. Notably, all engineered peptide complexes displayed substantially lower and more stable RMSD profiles compared with the WT, with none exceeding 2.0 Å at any point in the simulation. This clear separation between WT and MT peptides was consistent across all trajectories, indicating that the introduced mutations not only strengthened the protein–peptide interactions but also minimized structural fluctuations at the binding interface. The small, stable RMSD fluctuations for all MT complexes suggest limited conformational rearrangement, reflecting a high degree of structural rigidity and binding persistence. Regarding biological meaning, RMSD values in the range of 1–2 Å for a protein–peptide complex are generally considered to reflect strong structural stability and sustained binding, with little likelihood of unbinding or major conformational change during the simulation period. Although very low RMSD values can occasionally be artifacts arising from simulation restraints or force field limitations, in this study, positional restraints were only applied during the equilibration phase, and the production runs were performed with the ff19SB force field—a robust, widely accepted model for proteins and peptides. These results collectively demonstrate that the engineered peptide variants confer a significant improvement in the dynamic stability of the ML‐IAP complex relative to the WT sequence. The lower RMSD values observed for the MTs suggest a tighter and more persistent binding mode, which may be predictive of enhanced inhibitory efficacy in subsequent functional assays. This stability highlights the effectiveness of the rational peptide design strategy employed in this study.

Figure 8(a–d) Root‐mean‐square deviation (RMSD) profiles of ML‐IAP–peptide complexes over 200‐ns molecular dynamics simulation.

**Figure:**
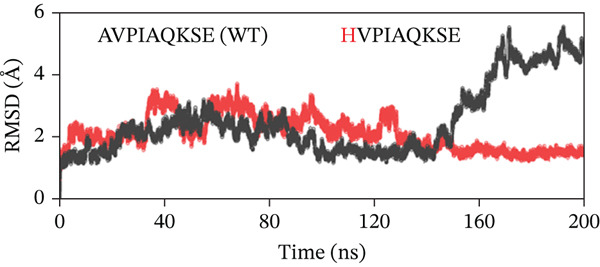
(a)

**Figure:**
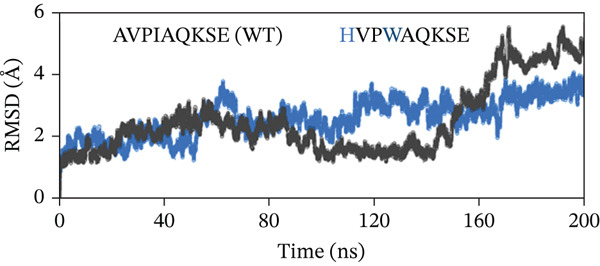
(b)

**Figure:**
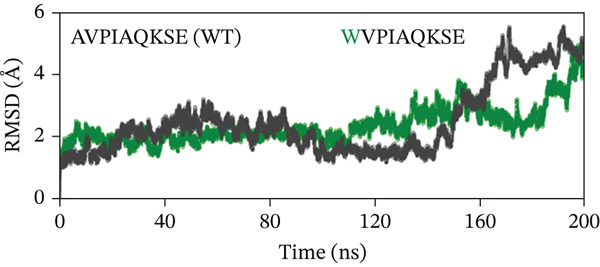
(c)

**Figure:**
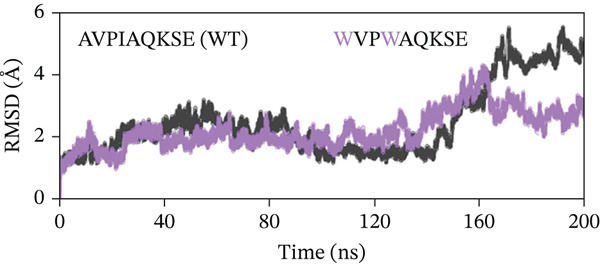
(d)

##### 3.4.4.2. Residual Flexibility Analysis.

To further investigate the local flexibility and dynamic behavior of the ML‐IAP protein upon binding to different peptide variants, RMSF analysis was performed on the backbone atoms of each complex over the course of the 200‐ns MD simulations (Figure 9). RMSF quantifies the average positional deviation of individual residues from their mean positions, providing insights into regions of structural rigidity and flexibility. The WT peptide complex (AVPIAQKSE) exhibited a characteristic RMSF profile, with higher fluctuations primarily observed at the N‐termini and C‐termini and within several surface‐exposed loops, in line with the intrinsic flexibility of these regions. Notably, binding of the HVPIAQKSE MT led to increased RMSF values at specific sites, particularly around Residues 30, 50, and 70. These residues are located within surface loops not directly involved in peptide binding or the functional core, indicating that the observed flexibility does not compromise the stability of essential structural or catalytic regions. The WVPIAQKSE MT showed an RMSF pattern largely comparable with that of the WT, suggesting a minimal alteration in overall protein flexibility. In contrast, the HVPWAQKSE double MT induced a more pronounced and widespread increase in RMSF, suggesting an overall enhancement in protein dynamics, which could correspond to a more adaptive or less rigid binding interaction. Conversely, the WVPWAQKSE double MT demonstrated the lowest RMSF values among all complexes, indicating increased structural rigidity and compactness of the protein upon binding this variant. Across all systems, localized increases in RMSF were primarily observed near Residues 30 and 70, corresponding to loop regions adjacent to the active site, likely reflecting local adaptations to accommodate the structural features of the engineered peptides. RMSF values below 1 Å indicate that the corresponding regions of the protein remain structurally stable and well‐ordered during the simulation, which is typically observed for residues involved in binding interfaces or secondary structural motifs. The prevalence of sub‐angstrom fluctuations in these complexes suggests that peptide binding does not induce significant destabilization or disorder in ML‐IAP, but rather supports the formation of stable, tightly bound complexes. Nonetheless, it should be acknowledged that such low RMSF values can sometimes result from simulation artifacts, including excessive restraint, limited equilibration, or force field biases. To mitigate these risks, all simulations in this study employed the ff19SB force field, positional restraints were limited to the equilibration phase, and long production trajectories were conducted to ensure robust sampling.

Figure 9(a–d) Root mean square fluctuation (RMSF) profiles of ML‐IAP–peptide complexes over 200 ns molecular dynamics simulation.

**Figure:**
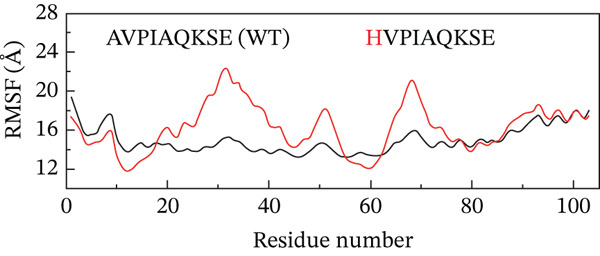
(a)

**Figure:**
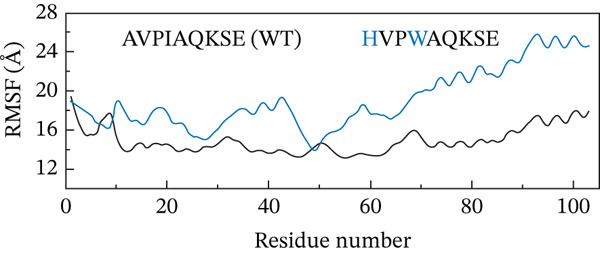
(b)

**Figure:**
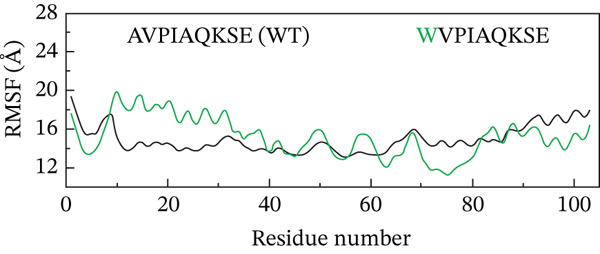
(c)

**Figure:**
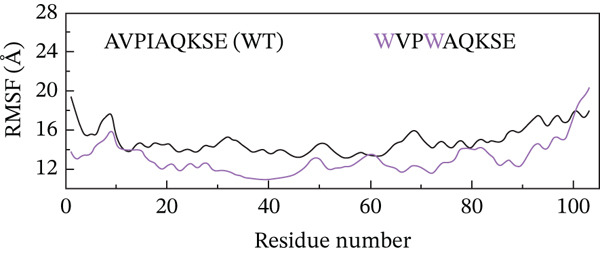
(d)

##### 3.4.4.3. Hydrogen Bond Analysis.

Hydrogen bond analysis was conducted to identify potential hydrogen‐bonding interactions within the protein structure. This analysis used specific distance and angle criteria to determine the presence of hydrogen bonds. The threshold for the donor–acceptor distance was set at 3.5 Å, whereas the angle between the donor, hydrogen, and acceptor was limited to 30°. This method ensures that only the most likely and relevant hydrogen‐bonding interactions are considered, providing insights into the structural stability and interaction dynamics of the protein. This analysis identified numerous hydrogen bonds throughout the protein structure. Concerning the H‐bonds formation, all of the examined peptide variants formed many more H‐bonds with the receptor as compared with the native peptide, signifying the higher potency and stronger binding of the examined peptides relative to the original version. Although the H‐bonds formed throughout the whole course of MD simulation, the last 50 ns witnessed a greater number of H‐bonds. The increased number and lifetime of these hydrogen bonds may suggest a mechanistic advantage in the form of prolonged residence time of the peptide within the binding pocket. This stabilization effect could be critical for maintaining an active binding conformation and enhancing the overall interaction efficiency between the MT peptide and the receptor. Notably, the double‐MT HVPWAQKSE was the best one in terms of H‐bonds formation and higher number of them, as illustrated in Figure 10.

Figure 10H‐bonds plot of the best peptide mutants and the original peptide (WT). (a) AVPIAQKSE (WT) and HVPIAQKSE. (b) AVPIAQKSE (WT) and HVPWAQKSE. (c) AVPIAQKSE (WT) and WVPIAQKSE. (d) AVPIAQKSE and WVPWAQKSE.

**Figure:**
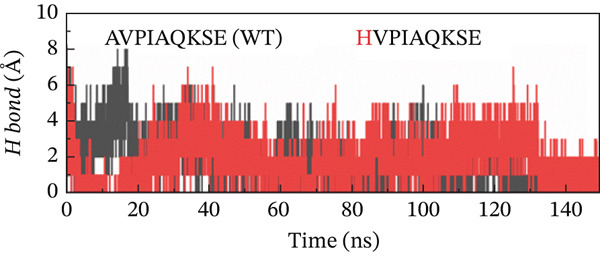
(a)

**Figure:**
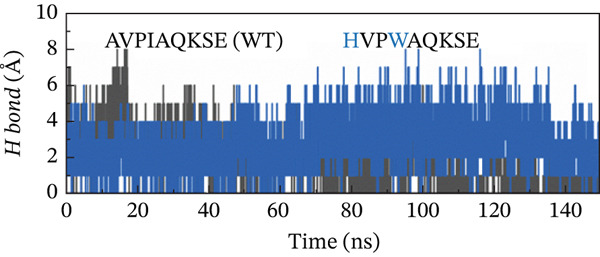
(b)

**Figure:**
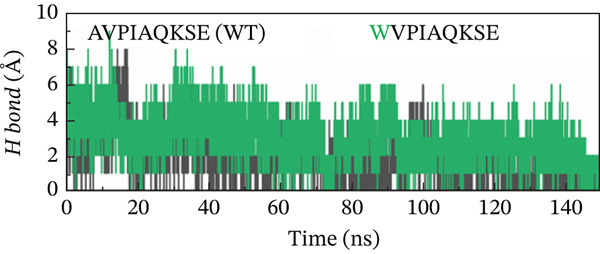
(c)

**Figure:**
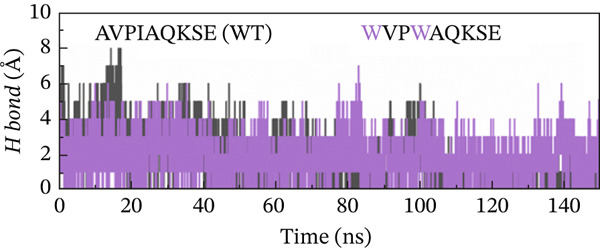
(d)

##### 3.4.4.4. Protein Compactness Investigation.

The Rg was used to assess the compactness of the protein structure before and after ligand binding (Figure 11). The Rg values for the original peptide (WT) and HVPIAQKSE were 13.32 and 13.45 Å, respectively. The binding of the peptide resulted in a slight increase in the Rg value, indicating that the peptide occupied space within the protein‐binding site. This binding also disrupted the tight packing of the protein secondary structure, leading to a slight increase in the Rg value (particularly from 50 to 150 ns). However, after the formation of a stable protein–peptide complex, the Rg value remained relatively steady with small fluctuations for the remainder of the simulation, averaging at 13.32 Å. In the case of the HVPIAQKSE system, the Rg continuously increased during the first 110 ns of the simulation, averaging between 14.0 and 13.11 Å. However, the Rg then stabilized, indicating a tight conformation of the protein‐HVPIAQKSE complex. The complex eventually reached a stable conformation with an average Rg value of 13.3 ± 0.0017 Å until the end of the simulation. Similarly, the WVPIAQKSE‐bound protein complex showed a minor expansion in the Rg value (13.20 ± 0.001 Å) with no deviations recorded during the 200‐ns simulation, suggesting a stable protein–peptide complex. Conversely, in the case of the WVPWAQKSE system, there was a constant increase in the Rg value during the last 110 and 200 ns of the simulation, reaching a maximum value of 14.15 ± 0.001 Å. These results collectively indicate a compact arrangement of the protein after peptide binding, supporting the establishment of a stable protein–peptide complex.

Figure 11(a–d) The radius of gyration (Rg) graph of original peptide (WT) and selected inhibitor–Mpro complexes during the 200‐ns MD simulation.

**Figure:**
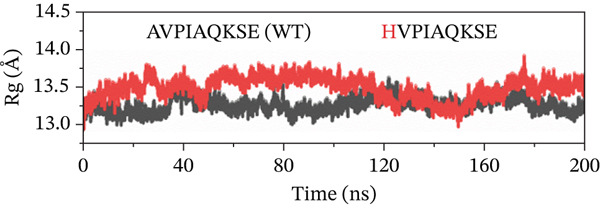
(a)

**Figure:**
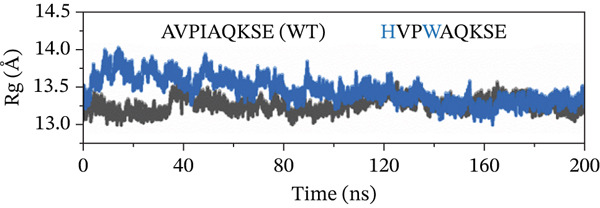
(b)

**Figure:**
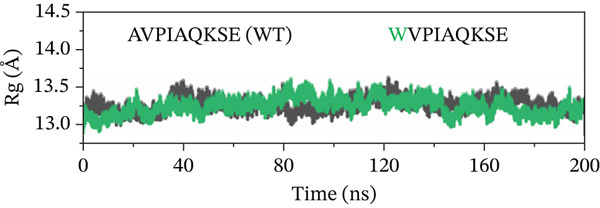
(c)

**Figure:**
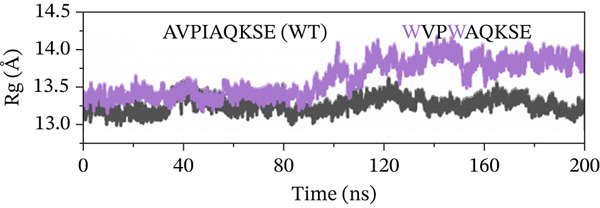
(d)

##### 3.4.4.5. Principal Component Analysis (PCA).

**Figure 12:**
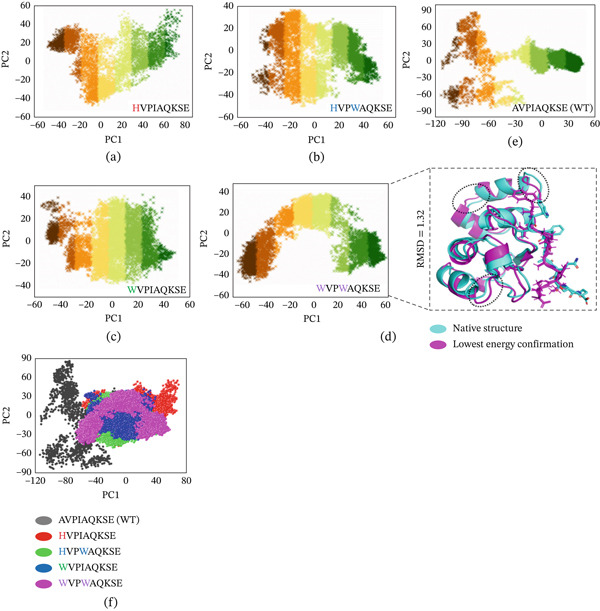
(a–f) The principal component analysis of original peptide (WT) and selected peptide inhibitor. The projection of the conformational is defined by the first two principal components: PC1 (plotted at the *x*‐axis) and PC2 (plotted at the *y*‐axis). Additionally, the native structure and lower energy conformation showed that this peptide binds strongly and alters the protein structural dynamics.

The motion of MD trajectories was analyzed, and the functional mobility of macromolecules was better understood by carrying out PCA, an unsupervised learning technique. Performing PCA on proteins in three‐dimensional space allows us to identify the directions in which proteins experience the most and least fluctuations. This analysis provides insights into the structural stability, flexibility, and fluctuation of essential regions of the protein. Large eigenvalues in the corresponding eigenvectors offer clues about the protein′s function, representing the sum of its coordinated movements. The Cpptraj package was employed to process the Cartesian coordinates of C atoms for 6000 snapshots of each simulation throughout the trajectory to conduct the PCA. The PCA of the WT and the selected peptide MTs is shown in Figure 12. In the WT, peptide AVPIAQKSE, the total contributed variance by the first eigenvectors to the total motions was reported to be −110 and +40 along the first principal component (PC1) and −80 and +70 along the second principal component (PC2) (Figure 12e), whereas in the cases of HVPIAQKSE and HVPWAQKSE, the variance by the three eigenvectors was observed to be −50 and +50 along PC1 and −50 and +55 along PC2, and −40 and +40 along PC1 and −50 and +30 along PC2, respectively (Figure 12a,b). Similarly, in the case of WVPIAQKSE, the variance by the three eigenvectors was observed to be (−60 and +50 along PC1 and −40 and +40 along PC2), whereas in the case of WVPWAQKSE , −40 and +40 along PC1 and −50 and +40 along PC2 (Figure 12c,d). Among all of the MTs, one single‐MT WVPIAQKSE as well as a double‐MT HVPWAQKSE were the best models in this attribute. In order to confirm the overall system confirmation, whether the systems explore the same conformational space or not, we computed combined PCA for all the system (Figure 12f). These findings conclude that these selected peptide inhibitors have the potential to bind strongly and alter the protein structural dynamics required for the protein′s normal function.

##### 3.4.4.6. Protein Structural Changes Analysis.

**Figure 13:**
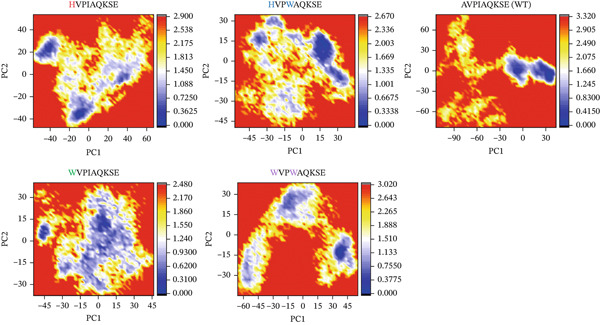
Principal component analysis (PCA) of WT and MTs. The first PC1 and second PC2 from the PCA of the backbone carbon were used.

The Free Energy Landscape (FEL) demonstrates the transition states between various conformations of WT and MT complexes over the trajectory time, utilizing PC1 and PC2. To comprehend the transition mechanism of MTs and WT complexes from metastable to native states, the first two eigenvectors were utilized to compute and visualize the FEL over the trajectory time. To better understand the structural evolution, low‐energy states were identified. A significant difference in FEL was observed between WT and MTs, as depicted by the colors in the plot (Figure 13). The prevalence of the color red in MTs suggests instability compared with WT. The highest transition states were observed in HVPIAQKSE, HVPWAQKSE, WVPIAQKSE, and WVPWAQKSE, indicating the impact of these residue mutations on protein binding. In contrast, WT exhibited two states separated by an energy barrier.

## 4. Discussion

The survival rate of metastatic melanoma was estimated to be less than 5 years. In reality, it is uncurable up to date [32]. As the early‐stage melanoma cells tend to be localized, the surgical operation is the primary option in hand. However, metastatic melanoma needs adjuvant therapy. This involves interferon alpha for patients having a tumor thicker than 1.5 mm. In addition to ipilimumab, nivolumab, and pembrolizumab [33], the main obstacle of these therapeutics lie in their low responsiveness ranging from 15% to 43% [34, 35]. Recently, the emergence of ACP greatly served the therapeutic options of different cancer types including melanoma. For instance, a melittin‐RADA32 hybrid peptide hydrogel linked to doxorubicin can recruit activated natural killer cells in the primary melanoma tumor, resulting in growth retardation. It also activates dendritic cells of draining lymph nodes and produces cytotoxic T‐cells against the remaining tumors [8, 36]. A tyrosinase‐related protein 2 melanoma antigen peptide nanovaccine combined with CpG adjuvant resulted in slow growth of the melanoma tumor [37]. Promising results were reported from gaining the FDA‐approval of gp100: 209‐217 (210 M)/Montanide ISA‐51/Imiquimod, which promoted many researchers from all over the world to investigate the potential of ACP [38]. Compared with small‐molecule inhibitors, peptides are generally more specific and selective, with fewer side effects. The present study is aimed at improving the ACP of ML‐IAP through computational peptide engineering so that it poses stronger binding and safer side effects. We first identified the hotspot residues that lie in the peptide–protein interface and participate in the noncovalent binding. These hotspots were then subjected to saturation mutagenesis using the mCSM‐PPI 2 server. The best of first generation (single MTs) outputs HVPIAQKSE and AVPWAQKSE with docking score of −161 and −159 kcal/mol via HPEPDOCK 2. We then merged the two MTs in order to get more enhanced binding, that is, substituting the first alanine residue with histidine along with replacing the fourth isoleucine with tryptophan (generated double MTs). HVPWAQKSE and WVPWAQKSE displayed stronger binding to the protein with docking scores of −167 and −173 kcal/mol compared with the original peptide (–125 kcal/mol). It is important to note that the binding affinity values reported in this study, as derived from the HPEPDOCK 2 docking scores, are not directly equivalent to experimentally determined binding free energies or dissociation constants (Kd). These computational scores are best interpreted for relative ranking of binding potential among peptide variants, rather than as absolute measures of affinity. To confirm and quantify the true binding affinities of the engineered peptides, further experimental assays such as surface plasmon resonance (SPR) or isothermal titration calorimetry (ITC) will be necessary. The second part is aimed at checking the safety profile of the enhanced peptides by checking their toxicity and allergenicity. We found that three of the single MTs and one of the double MTs exhibited toxicity issue, whereas none of the generated peptides displayed allergenicity. The allergenicity of the examined peptides can be tested in vitro using Local Lymph Node Assay (LLNA) and Peptide Reactivity Assays [3]. Likewise, cytotoxicity can be checked via MTT assay and RBC hemolytic assay [4].

In terms of conformational changes and dynamics, the generated variants displayed less conformational alterations and local motions. Previous reports show that less conformational and local changes indicate more compact configuration and more stability as inferred from PCA output [39–41]. Hence, the findings of the present study suggest that the produced peptides, both single and double MTs, are superior candidates as inhibitors to ML‐IAP and, thus, cancel the inhibition imposed by the apoptosis‐blocking protein. Consequently, the melanoma cells will undergo apoptosis (programmed cell death) and, thereby, most of the cancerous cells will die. Moreover, even patients with metastatic melanoma will benefit from this therapeutic option, and their potential survival rate will be improved [42]. Nonetheless, these results require the translation in the clinical setting via in vitro and in vivo experimentation.

## 5. Conclusion

The aim of this study is to improve the binding affinity and safety profile of the ML‐IAP nonapeptide AVPIAQKSE through in silico peptide engineering. The study employed saturation mutagenesis to identify the most promising amino acid substitutions with a positive impact on binding affinity. The lead candidates were docked to the receptor using HPEPDOCK 2, and their safety profiles were evaluated using the ToxIBTL and AllerCatPro 2 servers. The study identified HVPIAQKSE, WVPWAQKSE, and HVPWAQKSE as the best MTs, potentially superior to the original peptide in terms of binding affinity and safety profile (nontoxic and nonallergenic). These variants displayed increased flexibility, reduced conformational alterations and local motions, and a more compact configuration, suggesting greater stability compared with the reference peptide. Although the computational results are promising, further optimization and experimental validation are essential to address practical challenges such as peptide stability, proteolytic degradation, and limited cell permeability. Based on these computational analyses, these MTs warrant further validation in vitro as potential therapeutic options for melanoma ACPs.

## Author Contributions

**Haitham Ahmed Al-Madhagi and Muhammad Shahab**: performed conceptualized the study, overall guidance, and manuscript writing, software, and writing—original draft preparation. **Muhammad Shahab**: methodology, validation, and formal analysis; **Gamil Al-Madhagy**: data curation. **Zheng Guojun, Zunnan Huang, and Musaab Dauelbait**: supervised the project, approved, and submitted the final manuscript. **Gamal A. Shazly and Mohammed Bourhia**: validation, formal analysis, and funding acquisition.

## Funding

This work is financially supported by the Ongoing Research Funding Program (ORF‐2026‐1118), King Saud University, Riyadh, Saudi Arabia.

## Ethics Statement

The authors have nothing to report.

## Conflicts of Interest

The authors declare no conflicts of interest.

## Data Availability

All structural data analyzed in this study were obtained from the publicly available Protein Data Bank (PDB; ID: 1OXQ). No new experimental, clinical, or structural datasets were generated during this work. The data will be available upon request from the authors.
